# Immunomodulation Induced During Interferon-α Therapy Impairs the Anti-HBV Immune Response Through CD24^+^CD38^hi^ B Cells

**DOI:** 10.3389/fimmu.2020.591269

**Published:** 2020-12-23

**Authors:** Binqing Fu, Dongyao Wang, Xiaokun Shen, Chuang Guo, Yanyan Liu, Ying Ye, Rui Sun, Jiabin Li, Zhigang Tian, Haiming Wei

**Affiliations:** ^1^Division of Molecular Medicine, Hefei National Laboratory for Physical Sciences at Microscale, The CAS Key Laboratory of Innate Immunity and Chronic Disease, School of Life Sciences, University of Science and Technology of China, Hefei, China; ^2^Institute of Immunology, University of Science and Technology of China, Hefei, China; ^3^Department of Infectious Diseases, The First Affiliated Hospital of Anhui Medical University, Hefei, China

**Keywords:** anti-virus function, immunomodulatory effects, CD24^+^CD38^hi^ B, chronic hepatitis B virus infection, Peg-IFNα-2b

## Abstract

Type I interferon is widely used for antiviral therapy, yet has yielded disappointing results toward chronic HBV infection. Here we identify that PEG-IFNα-2b therapy toward persistent infection in humans is a double-edged sword of both immunostimulation and immunomodulation. Our studies of this randomised trial showed persistent PEG-IFNα-2b therapy induced large number of CD24^+^CD38^hi^ B cells and launched a CD24^+^CD38^hi^ B cells centered immunosuppressive response, including downregulating functions of T cells and NK cells. Patients with low induced CD24^+^CD38^hi^ B cells have achieved an improved therapeutic effect. Specifically, using the anti-CD24 antibody to deplete CD24^+^CD38^hi^ B cells without harming other B cell subsets suggest a promising strategy to improve the therapeutic effects. Our findings show that PEG-IFNα-2b therapy toward persistent infection constitutes an immunomodulation effect, and strategies to identifying the molecular basis for the antiviral versus immunomodulatory effects of PEG-IFNα-2b to selectively manipulate these opposing activities provide an opportunity to ameliorate anti-virus immunity and control viral infection.

## Introduction

Human hepatitis B virus (HBV) establishes persistent infection and thus constitutes a major health burden worldwide (1–4). More than 240 million people are chronically infected with HBV globally, and 780,000 people die each year from HBV-associated liver diseases (5). Peg-IFNα-2b, which mediates both immunomodulatory and direct antiviral effects, is currently widely utilized for the treatment of HBV (6, 7). Interferon-α (IFNα) signaling occurs upstream of hundreds of inflammatory genes and leads to a hyper-activated immune response. These responses include activation of the adaptive and innate immune systems, and increased production of antiviral proteins capable of suppressing viral replication and promoting viral clearance (3, 8–10). However, PEG-IFNα-2b yields disappointing therapeutic effects when used to treat chronic HBV infection. Actually, Hepatitis B e antigen (HBeAg)-positive CHB patients who received PEG-IFNα-2b, PEG-IFNα-2b plus lamivudine, or lamivudine alone demonstrated only a 32, 27, or 19% rate of HBeAg seroconversion, respectively (6). The explanation for these results remains unknown.

Recently, reports about persistent infections using lymphocytic choriomeningitis virus (LCMV) infection in mice revealed that Type I Interferon potentially initiates an immunosuppressive program that represses antiviral immunity and facilitates persistent infection. For example, in a model of persistent LCMV, the blockade of IFN-I signaling reduced immune system activation, decreased the levels of negative immune regulatory molecules, and promoted viral clearance and infection control (11, 12). Moreover, a rapid depletion of virus-specific B cells have been documented and this deletion can be completely reversed by blockade of Type I interferon (IFN-I) signaling, associated with suboptimal antibody responses (13–15). It would be very interesting to explore whether IFN-I induced the immunomodulatory effect also exist in virus infected human.

In HBeAg-negative chronic patients, the absolute number of HBV-specific CD8^+^ T cells was strikingly reduced on PEG-IFNα-2b therapy whereas numbers of CD56^bright^ NK cell increased, showing differential boosting of innate and adaptive antiviral responses (4, 16).Also in HBeAg-negative chronic patients, PEG-IFNα-2b therapy showed a major impact on peripheral B cell subsets and a complete remodeling of the B cell compartment (17). In HBeAg-positive chronic patients, PEG-IFNα-2b has shown to induce a distinct and rapid up-regulation of IFN signaling pathway that coincided with the up-regulation of the frequency of proliferating NK and activated total CD8^+^ T cells in the first 14 days of IFN-α treatment (18). However, whether PEG-IFNα-2b therapy can induce an immunomodulatory program in chronic infected HBeAg-positive patients during the whole 48 weeks of PEG-IFNα-2b treatment has not yet been determined.

D24 is a small glycosyl phosphoinositol anchored protein that is able to provide costimulatory signals to T cells and the CD24-Siglec-G pathway has been identified to selectively suppresses the immune response to Danger-associated molecular patterns (DAMPs) (19). CD24 can be the dominant innate immune checkpoint and novel “Don’t Eat Me” signal that promotes ovarian cancer and breast cancer immune escape (20). CD24 has also been identified as a marker of regulatory B (Breg) cells. CD24^+^CD38^hi^ Breg cells inhibit the differentiation of Th1 and Th17 cells, and suppress effector CD4^+^ and CD8^+^ T cells *via* the release of IL-10 (21–25). CD24 polymorphisms affect the risk and progression of chronic HBV infected patients. Targeted mutation of CD24 drastically reduced the size of spontaneous liver cancers in HBV transgenic mice (26). It has been reported the existence of IL-10-secreting CD24^+^CD38^hi^ B cells in HBV patients (27); however, the dynamic change and the function of these CD24^+^CD38^hi^ B cells during PEG-IFNα-2b therapy has not been uncovered.

To determine whether Peg-IFNα-2b therapy causes immunomodulatory effects, randomized clinical trial were conducted including 92 naive HBeAg-positive CHB patients. Patients were divided into two groups, one receiving Peg-IFNα-2b alone and one receiving Peg-IFNα-2b in combination with adefovir-dipivoxil, in order to simulate patients undergoing treatment with nucleoside analog (NUC). Samples were characterized at multiple time points through the whole 48 weeks of PEG-IFNα-2b therapy and also 24 weeks of follow-up. The data revealed a new mechanism in which Peg-IFNα-2b therapy during persistent infection in humans launches a CD24^+^CD38^hi^ B -centered immunomodulatory program. This mechanism counteracts the antiviral ability of the immune system in patients with chronic HBV infection.

## Materials and Methods

### Ethics Statement

This multi-centered, randomized, open-label research study was conducted in accordance with the guidelines of China’s regulatory requirements, the Declaration of Helsinki and the Principles of Good Clinical Practice. This trial was approved by the local Ethics Board of the First Affiliated Hospital of Anhui Medical University with the clinical trial registration number ChiCTR-TRC-12002226 (http://www.chictr.org.cn/index.aspx). The detail about this Clinical Trial protocol has been showed in the **Supplementary Materials**. All patients involved were HBV patients who had not undergone prior antiviral or immunomodulatory treatment, and each provided written informed consent. Peripheral blood samples from healthy donors were obtained from the Blood Center of Anhui Province. Ethical approval was obtained from the Ethics Committee of the University of Science and Technology of China.

### Patients and Human Samples

**Figure 1:**
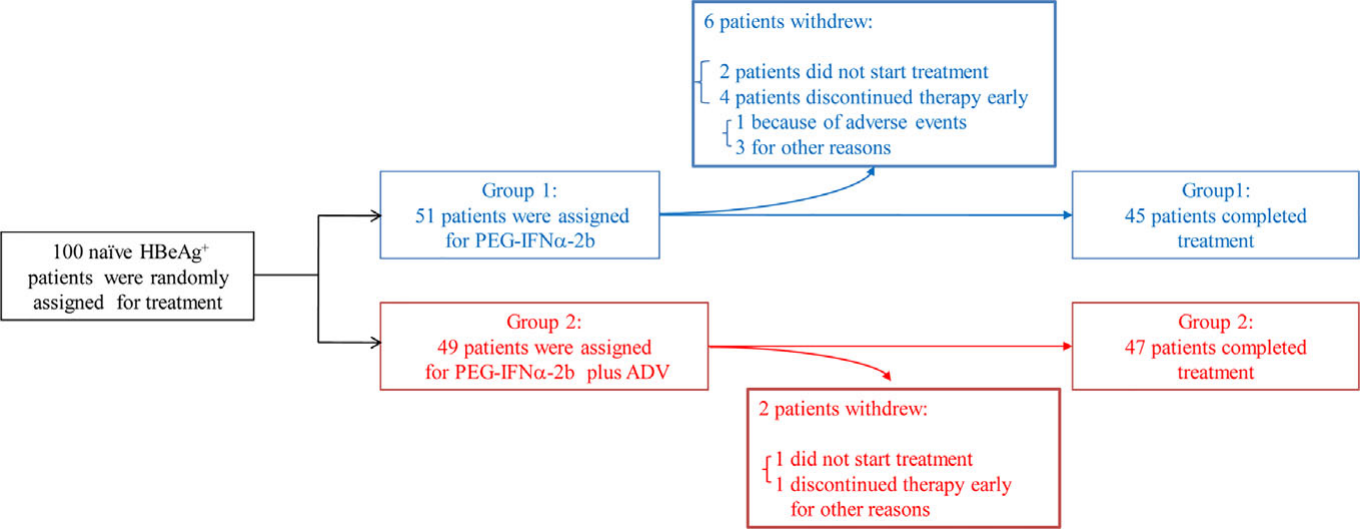
Patients through the trial. One hundred patients that had been positive for HBeAg and hepatitis B surface antigen (HBsAg) for longer than 6 months and had elevated serum alanine transaminase (ALT) (>2 × ULN and <10 × ULN) and detectable baseline serum HBV DNA (>2 × 104 IU/ml) on at least two occasions have been involved into this clinical trial. Out of 100 HBeAg positive patients, 92 were completed the final Peg-IFNα-2b treatment. Randomized clinical trial were conducted including these 92 naive HBeAg-positive CHB patients. Patients were divided into two groups, one receiving Peg-IFNα-2b alone and one receiving Peg-IFNα-2b in combination with adefovir-dipivoxil in order to simulate patients undergoing treatment with nucleoside analog (NUC).

The included patients had been positive for HBeAg and hepatitis B surface antigen (HBsAg) for longer than 6 months and had elevated serum alanine transaminase (ALT) (>2 × ULN and <10 × ULN) and detectable baseline serum HBV DNA (>2 × 10^4^ IU/mL) on at least two occasions. Those who had liver cirrhosis, antibodies against HCV, hepatitis D virus, or HIV, or other acquired or inherited causes of liver disease were excluded. The patients were randomly assigned into one of two groups to receive Peg-IFNα-2b (1.5 µg/kg/week, PegIntron, Schering-Plough, Kenilworth, NJ, USA) alone or in combination with adefovir-dipivoxil (ADV) (10 mg/day, Hepsera, Gilead Sciences, Foster City, CA, USA) for 48 weeks with 24 weeks of follow-up (**Table S1**). An HBeAg seroconversion was defined as a patient with HBeAg loss (COI < 1.0) and seroconversion to anti-HBeAg at week 72 (**Table S2**). According to the European Association for the Study of the Liver guidelines (28), sustained response patients consisted of 17 responders in these 92 patients defined as persistently undetectable HBeAg and a result of HBV DNA <2,000 IU/ml, with the development of antibodies to HBeAg (anti-HBe) (29). Out of 100 HBeAg positive patients, 92 were completed the final PEG-IFNα-2b treatment (**Figure 1**). NUC-alone patients are also patients had been positive for HBeAg and hepatitis B surface antigen (HBsAg) for longer than 6 months but voluntarily choose to use NUC medicines but not PEG-IFNα-2b therapy. The samples of NUC-alone patients were collected at 6 months or 9 months after the NUC therapy.

Other detailed materials and methods are available in the **Supplementary Material**.

## Results

### CD24^+^CD38^hi^ B Cells Are Induced Significantly During Peg-IFNα-2b Therapy

**Figure 2:**
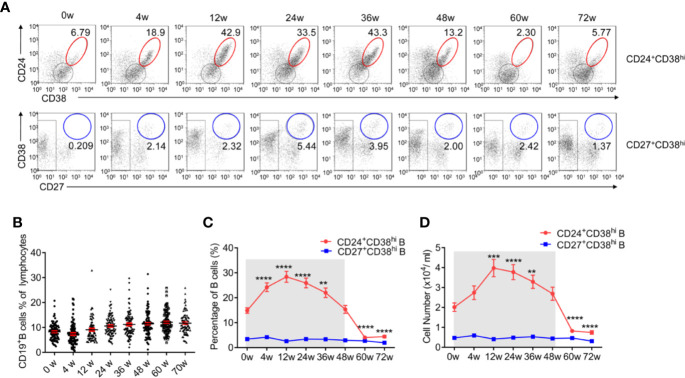
CD24^+^CD38^hi^ B cells increase significantly in HBV patients during Peg-IFNα-2b therapy. Naive HBeAg-positive CHB patients were divided into two groups, one receiving Peg-IFNα-2b alone and one receiving Peg-IFNα-2b in combination with adefovir-dipivoxil, in order to simulate patients undergoing treatment with NUCs. Data were quantified from blood samples during routine visits to hepatitis clinics (weeks 0, 4, 12, 24, 36, and 48) and during follow-up (weeks 60 and 72). **(A)** Representative density plots showing CD24 and CD38 expression as well as CD27 and CD38 expression on gated CD19^+^B cells at each time point during Peg-IFNα-2b therapy. **(B)** Percentage analysis of CD19^+^B cells from HBV patients at 0-72w during Peg-IFNα-2b therapy. n = 75–90. Unpaired t test. **(C)** Percentage analysis of CD24^+^CD38^hi^ B cells and CD27^+^CD38^hi^ B cells from HBV patients at 0-72 week during PEG-IFNα-2b therapy. n = 47. Unpaired t test. **(D)** Cell numbers of CD24^+^CD38^hi^B cells and CD27^+^CD38^hi^ B cells from HBV patients at 0-72w during PEG-IFNα-2b therapy. n = 47. Unpaired t test. Significant differences are shown in **(C, D)** indicating differences compared with the data at 0w. Mean ± SEM, **P < 0·01, ***P < 0·005, ****P < 0·0001.

Similar to previous studies (6), 30.4% of HBV patients in our study underwent HBeAg seroconversion. There was no significant difference (p = 0·501) between Group 1, the Peg-IFNα-2b group (26.7%), and Group 2, the Peg-IFNα-2b + adefovir-dipivoxil group (34.0%) (**Figure 1** and **Tables S1** and **S2**). No significant change of the total CD3^-^CD19^+^B cells during PEG-IFNα-2b therapy (**Figure 2B**). To investigate whether Peg-IFNα-2b therapy induced immunomodulatory effects, analysis of a variation among B cell subsets in HBV patients *in vivo* including CD24^+^CD38^hi^ B cells and CD27^+^CD38^hi^ B cells were done in the whole Peg-IFNα-2b therapy. These studies showed that the percentage and cell number of CD24^+^CD38^hi^ B cells increased rapidly after Peg-IFNα-2b was administered, remained stable at high levels during therapy, and decreased when Peg-IFNα-2b therapy was discontinued at week 48 (**Figures 2A, C, D**). Furthermore, CD24^+^CD38^hi^ B cells increased in patients from both Groups 1 and 2 (**Figures S1A, B**). To further exclude the changes of CD24^+^CD38^hi^ B cells is for the reason that CHB patients have hepatic flares time to time, negative control groups with no-treatment and NUC-alone therapy were also measured. Both negative control groups showed a low percentage of CD24^+^CD38^hi^ B cells (**Figures S1C, D**). On the other hand, previous researches showed that a rapid depletion of antibody-producing mature B cells correlated with IFNα signaling (13–15). In persistent HBV patients, there were no significant changes in the CD27^+^CD38^hi^ subset during Peg-IFNα-2b therapy, suggesting plasma B cells are always at low levels during Peg-IFNα-2b therapy (**Figures 2A, C, D**). Thus, persistent HBV patients have increased CD24^+^CD38^hi^ B cells during Peg-IFNα therapy.

### Peg-IFNα-2b Induced CD24^+^CD38^hi^ B Cells Secret IL-10

**Figure 3:**
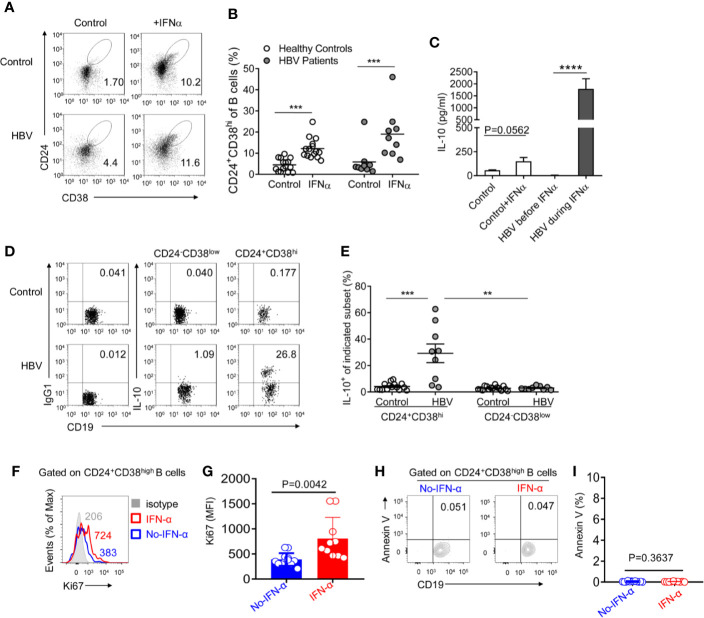
CD24^+^CD38^hi^ B cells are induced under Peg-IFNα-2b and produce plenty of IL-10. **(A**, **B)** PBMCs from HBV patients and healthy controls were cultured in complete RPMI medium 1640 with or without human Peg-IFNα-2b (100 ng/ml) for 36h. Representative density plots **(A)** and percentage analysis **(B)** showing CD24 and CD38 expression on gated B cells from HBV patients and healthy controls. n = 15 (healthy controls) and 9 (HBV patients). Paired t test. **(C)** ELISA analysis of IL-10 secretion in cell-free supernatants of cultured PBMCs from HBV patients before or during Peg-IFNα-2b therapy and healthy controls with or without re-stimulation by Peg-IFNα-2b (25 ng/ml) during a 32-h culture. PBMC from HBV patients were collected before PEG-IFNα-2b therapy or at 12-36 weeks after the start of Peg-IFNα-2b therapy. **(D)** Representative density plots and **(E)** percentage analysis of IL-10 expression on gated CD24^+^*CD38^hi^* B cells and CD24^-^CD38^low^ mature B cells from HBV patients and healthy controls. These B cells were from freshly isolated PBMC in HBV patients and healthy controls. After 4 h of stimulation with PMA and ionomycin in the presence of monensin, intracellular flow cytometry staining was done and gated as CD19^+^CD3^-^ cells. n = 15 (healthy controls) and 9 (HBV patients). Paired t-tests were used for the analysis in subsets from the same patient. Data are presented as Mean ± SEM, **P < 0·01, ***P < 0·005, ****P < 0·0001. **(F)** Flow cytometry analysis of Ki67 expression on the CD24^+^CD38^high^ B cells from PBMC of HBV patients without (blue) or with (red) PEG-IFNα-2b therapy for 12-24 weeks. **(G)** Statistics calculated by the MFI of Ki67 from PBMC of HBV patients without (blue, n = 12) or with (red, n = 10) PEG-IFNα-2b therapy for 12-24 weeks. Unpaired t test. **P < 0.01; ***P < 0.001; ****P < 0.0001. Data are presented as mean ± SD. **(H)** Flow cytometry analysis of Annexin V expression on the CD24^+^CD38^high^ B cells from PBMC of HBV patients without (blue) or with (red) PEG-IFNα-2b therapy for 12–24 weeks. **(I)** Statistics calculated by the percentage of Annexin V^+^ Breg cells from PBMC of HBV patients without (blue, n = 12) or with (red, n = 10) PEG-IFNα-2b therapy for 12–24 weeks. Unpaired t test. **P < 0.01; ***P < 0.001; ****P < 0.0001. Data are presented as mean ± SD.

Pro-inflammatory cytokines such as IFNα plays a pivotal role in B cell differentiation (25, 30–32). Thus, to determine whether Peg-IFNα-2b therapy increases the CD24^+^CD38^hi^ B cell population, Peripheral Blood Mononuclear Cells (PBMCs) from healthy donors and HBV patients were first cultured respectively through Peg-IFNα-2b stimulation (100 ng/ml) *in vitro*, which concentration was referred from previous researches (33). The percentage of CD24^+^CD38^hi^ B cells was significantly upregulated in both healthy donors and HBV patients when cells were cultured with Peg-IFNα-2b, indicating that IFNα stimulation induces the CD24^+^CD38^hi^ B cell population (**Figures 3A, B**). Interestingly, the frequency of CD24^+^CD38^hi^ B cells in PEG-IFNα-2b -treated HBV patient group were higher than those in PEG-IFNα-2b -treated healthy donor group, which is probably due to PBMC cells from HBV patient group were previously stimulated by Peg-IFNα-2b *in vivo*. To exclude the possibility that PEG-IFNα-2b treatment had a variable impact of the viability of diverse B cell subsets, and therefore may modulate the frequency of B cell subsets, the impact of PEG-IFNα-2b on survival of different B cell subsets was measured. The number of CD24^+^CD38^hi^ B cells from healthy controls was significantly increased. CD24^+^CD38^hi^ B subset from HBV patients were also the most variable subset compared with the other two subsets (**Figure S2**).

It has been reported the existence of IL-10-secreting B cells in HBV patients (27), however whether these IL-10-secreting B cells induced during PEG-IFNα-2b therapy has not been uncovered. PBMC were collected from HBV patients at 0 weeks or 12–36 weeks after the start of Peg-IFNα-2b therapy and cultured without Peg-IFNα-2b for 32 h. PBMCs collected from healthy donors were cultured with or without Peg-IFNα-2b (25 ng/ml) for 32 h. Much higher levels of IL-10 in PBMC cell supernatants from HBV patients during Peg-IFNα-2b therapy have been identified compared to the other groups (**Figure 3C**). To verify whether there are also other cells in the co-culture might produce IL-10, we have tested PBMC from HBV patients during Peg-IFNa therapy, Compared to non-B cells lymphocytes, including T cells, NK cells, B cells are the main source of IL-10 secreting (**Figure S3**). To uncover which subset is the main source of IL-10 in HBV patient during Peg-IFNα-2b therapy, B cells from freshly isolated PBMC of HBV patients and also healthy controls were analyzed. Data revealed that a higher percentage of CD24^+^CD38^hi^ B cells secreting IL-10 compared with other B cell subsets, suggesting the potential of these CD24^+^CD38^hi^ B cells to become IL-10-producing CD24^+^CD38^hi^ Breg cells (**Figures 3D, E**). To analyze whether PEG-IFNα-2b could induce expansion of these HBV-driven Bregs or prevent their death, we isolated PBMC from CHB patients with PEG-IFNα-2b treatment during 12–24 weeks, and CHB patients without PEG-IFNα-2b treatment. We tested the level of Ki67 expression and the percentage of apoptotic cells in Breg cells. The results showed that PEG-IFNα-2b might induce expansion of these HBV-driven Bregs (**Figures 3F–I**).

To identify whether these Breg cells however release other immunosuppressive cytokines such as IL-4, IL-4 expressions analysis have been done in HBV patient during PEG-IFNα-2b therapy. Our data showed that B cells from these HBV patients did not secret IL-4. In some patients, IL-4 has been identified to secrete from non-B cells but not B cells (**Figure S4**). Increase numbers of immature B cells might be a compensatory mechanism with accelerated emigration from BM or spleen. To demonstrate whether increase numbers of immature B cells is a compensatory mechanism with accelerated emigration from BM or spleen, we tested the circulating levels of BAFF, CXCL12, and CXCL13 of HBV patients at 12–36 weeks after the start of PEG-IFNα-2b therapy by ELISA. It seems CXCL12 has a positive correlation with Breg cells (**Figure S5**).

### Patients With Fewer CD24^+^CD38^hi^ B Cells Exhibit Improved Therapeutic Effects

**Figure 4:**
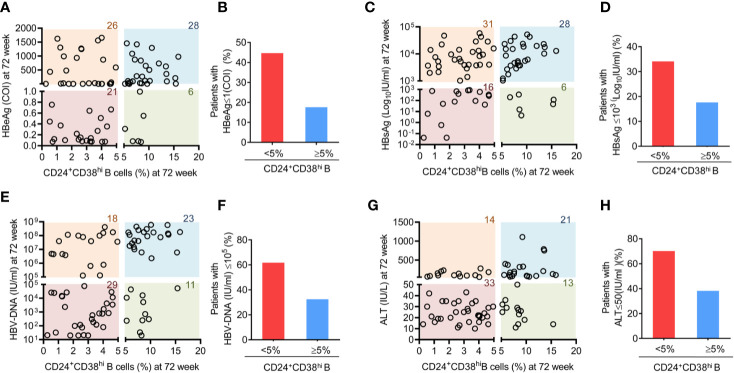
Patients with fewer CD24^+^CD38^hi^ B cells have an improved therapeutic effect. **(A)** Analysis between the percentage of CD24^+^CD38^hi^ B cells in patients at 72 week and HBeAg expression at 72 weeks after the start of Peg-IFNα therapy. n = 81. **(B)** Percentage analysis of SRs among patients with fewer CD24^+^CD38^hi^ B cells (<5%) group and more CD24^+^CD38^hi^ B cells (≥5%) group. SR (seroconversion responder) was defined as the HBeAg seroconversion (COI < 1.0) at 72 weeks after the start of PEG-IFNα-2b therapy. **(C, E, G)** Analysis between the percentage of CD24^+^CD38^hi^ B cells in patients at 72 week and HBsAg, HBVDNA and ALT expression at 72 weeks after the start of Peg-IFNα-2b therapy. n = 81. **(D, F, H)** Percentage analysis of patients with lower expression of HBsAg, HBVDNA and ALT expression at 72 weeks after the start of Peg-IFNα-2b therapy in fewer CD24^+^CD38^hi^ B cells (<5%) group and more CD24^+^CD38^hi^ B cells (≥5%) group. n = 81. Each circle in the Figure represents the data from a patient. The number marked on each color lump represent the sum number of patients.

As CD24^+^CD38^hi^ B cells increased dramatically during Peg-IFNα-2b therapy, we further investigated whether these CD24^+^CD38^hi^ B cells are relevant for clinical outcomes. CD24^+^CD38^hi^ B cells are found at low frequencies (<5% of B cells) in normal healthy human controls (34). Depending on whether the percentage of CD24^+^CD38^hi^ B cells in HBV patients at 72 weeks after the start of PEG-IFNα-2b therapy are higher than that in the normal healthy controls or not, patients were classified into the more CD24^+^CD38^hi^ B cell group (CD24^+^CD38^hi^ B cells ≥ 5% of B cells) or the fewer CD24^+^CD38^hi^ B cell group (CD24^+^CD38^hi^ B cell <5% of B cells). These groups were then analyzed for the clinical outcomes of PEG-IFNα-2b therapy. Among all the 81 patients that have tested both CD24^+^CD38^hi^ B cells phenotype and HBV related serum analysis, 44.68% percent of patients in the fewer CD24^+^CD38^hi^ B cell group underwent HBV seroconversion (HBeAg < 1.0 COI) compared with only 17.6% HBV seroconversion in the more CD24^+^CD38^hi^ B cell group (**Figures 4A, B**). Furthermore, we compared HBsAg, HBV DNA and ALT between patients in fewer CD24^+^CD38^hi^ B cell group and more CD24^+^CD38^hi^ B cell group, and the results indicated that patients in fewer CD24^+^CD38^hi^ B cell group may have higher probability to obtain satisfactory therapy results (**Figures 4C–H**). No significant differences between patients from fewer CD24^+^CD38^hi^ B cell group and more CD24^+^CD38^hi^ B cell group in HBeAg, HBsAg, HBV DNA, and ALT before Peg-IFNα-2b therapy (**Figure S6**). Thus, our data revealed that CD24^+^CD38^hi^ B cells have negative correlation with the therapeutic effects.

### CD24^+^CD38^hi^ B Cell-Centered Immunosuppressive Response by Inhibiting Anti-Virus Response

**Figure 5:**
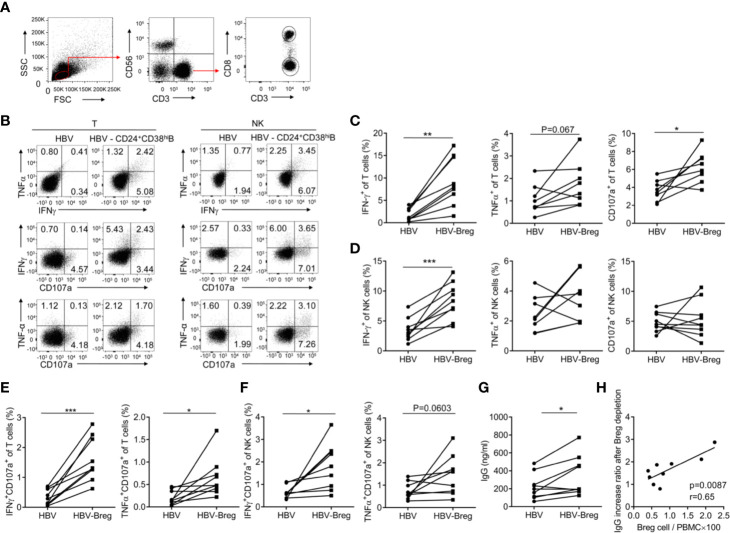
CD24^+^ CD38^hi^ B cells inhibit the anti-virus functions of T cells and NK cells. PBMCs from HBV patients at 12-36 weeks after the start of PEG-IFNα-2b therapy were cultured in 24-well ﬂat-bottom plates at a density of 1–2 × 106 cells per well in a complete RPMI medium 1640 with 10% fetal bovine serum plus streptomycin and penicillin as well as IL-2 (100 U/ml). PBMC from peripheral blood mononuclear cells of CHB patients with or without CD19+CD24+*CD38^hi^* B cells were sorted and the purity of depleting CD19+CD24+*CD38^hi^* B cells was determined to be >95% by post-purification FACS analysis. PBMC with or without CD19+CD24+*CD38^hi^* B cells were re-stimulated with 50 μg/ml of HBsAg (HyTest, 8HS7ay) for 72 h and cytokines were measured by intracellular staining. **(A)** The gating strategy for analyzing T- and NK cells. **(B)** Representative density plots of IFN*γ*, TNFα and CD107a expression on gated CD3^+^T cells or CD56^+^ CD3^-^NK cells from HBV patients with or without CD24^+^ CD38^hi^B cells. **(C**, **D)** Percentage analysis of IFN*γ*, TNFα and CD107a expression on gated CD3^+^T cells or CD56^+^NK cells from HBV patients with or without CD24^+^ CD38^hi^B cells (Breg). n = 8 in each group. Paired t-tests. **(E**, **F)** Percentage analysis of IFN*γ* ^+^CD107a^+^, TNFα^+^CD107a^+^ cells in gated CD3^+^T cells or CD56^+^CD3^-^NK cells from HBV patients with or without CD24^+^ CD38^hi^B cells (Breg). Paired t test. **(G)** Analysis of IgG secretion from HBV patients with or without CD24^+^CD38^hi^B cells. Paired t test. PBMC with or without CD19^+^CD24^+^CD38^hi^ B cells (Breg) were re-stimulated with 50 μg/ml of HBsAg for 72 h and cell-free supernatants of cultured PBMCs in each group were measured IgG by ELISA. **(H)** The IgG increase ratio (IgG level in PBMC after Breg depletion/IgG level in PBMC before Breg depletion) is positive correlated with the ratio between Breg cells and whole PBMC from HBV patients (Breg cell number/PBMC cell number×100). n = 9. Mean ± SEM, *p < 0·05, **P < 0·01, ***P < 0·005.

Our previous study has showed that increased NKp30 expression and degranulation on NK cells correlated with clinical outcomes during Peg-IFN-α-2b therapy (29). However, the number of NKp30^+^NK cell increased for the first 6 month but decreased for the rest of the therapy. Given the close association between CD24^+^CD38^hi^ B cells and clinical outcomes, the effects of CD24^+^CD38^hi^ B cells on immune cells function was further examined. To indicate whether the CD24^+^CD38^hi^ B cells have an inhibitory effect toward T cells and NK cells, high purity of PBMC with or without CD24^+^CD38^hi^B cells from HBV patients were sorted and re-stimulated with 50 μg/ml of HBsAg for 72 h. Cytokines were measured by intracellular staining. It has been shown that IFN*γ*, TNFα and CD107a positive T cells were significantly increased in CD24^+^CD38^hi^B cells depleted group (**Figures 5A, B, D**), suggesting CD24^+^CD38^hi^ B cells impaired anti-virus effect in T cells. It is interesting that IFN*γ*^+^NK cells, IFN*γ*^+^CD107a^+^NK cells were also significantly increased in CD24^+^CD38^hi^B cells depleted group compared with non-depleted group under the same culture condition (**Figures 5A, C, E**). These data showed that these CD24^+^CD38^hi^ B cells inhibit the anti-virus immune functions of T cells and NK cells. To further investigate the effect of the CD24^+^CD38^hi^ B cells on plasma cell function, antibody secretion were investigated from HBV PBMC stimulated by HBV specific antigens with or without CD24^+^CD38^hi^B cells. These data showed that after depleted CD24^+^CD38^hi^ B cells, HBV PBMC have higher IgG secretion (**Figure 5F**). To verify whether an increased IgG production has correlation with the percentage of CD24^+^CD38^hi^B cells, the correlation between CD24^+^CD38^hi^B cells and IgG increasing rate after CD24^+^CD38^hi^B cells have been analyzed. The IgG increase ratio (IgG in PBMC after CD24^+^CD38^hi^B cells depletion/IgG in PBMC before CD24^+^CD38^hi^B cells depletion) is positive correlated with the ratio between CD24^+^CD38^hi^B cells and whole PBMC from HBV patients (CD24^+^CD38^hi^B cells number/PBMC cell number) (**Figure 5G**). These data show that the CD24^+^CD38^hi^ B cells have an inhibitory effect toward T cells, NK cells and also IgG secretion.

**Figure 6:**
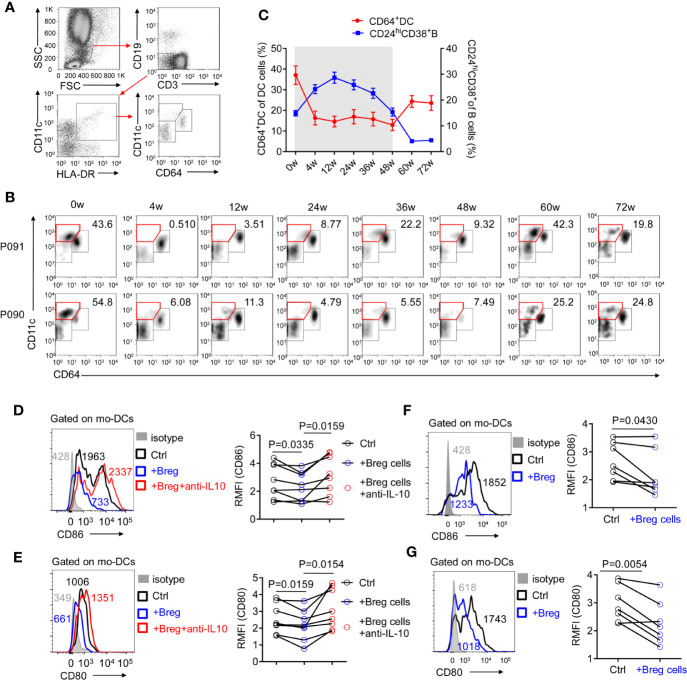
CD24^+^ CD38^hi^ B cells are negatively correlated with monocyte-derived DC cells. **(A)** Representative density plots of the gating strategy used for CD64^+^DC cells. **(B)** Representative density plots of CD11c and CD64 expressions on gated DC cells from HBV patients at each time point during PEG-IFNα-2b therapy. **(C)** Percentages analysis showing that CD24^+^CD38^hi^ B cells are negatively correlated with CD64^+^DC cells. n = 47. Mean ± SEM. **(D**–**G)** Mo-DCs were cultured in 48-well ﬂat-bottom plates at a density of 0.5-1.0 × 10^5^ cells per well in a complete RPMI medium 1640 (Gibco, Grand Island, NY, U.S.A.) with 10% fetal bovine serum (HyClone, Logan, UT, U.S.A.) plus streptomycin and penicillin. Equal number of Breg cells were then added to the culture wells, with or without 3 μg/ml of anti-IL-10 (biolegend; #501427) for 24 h. **(D, E)** Left: mo-DCs were co-cultured with Breg cells (blue) or with Breg cells and 3 μg/ml of anti-IL-10 (red), stained with CD86 **(D)** or CD80 **(E)**, and analyzed by flow cytometry. Right: The relative mean fluorescence intensity (RMFI) of n = 8 patients are shown. (**(F, G)** Left: mo-DCs were co-cultured with Breg cells (blue) in transwell system, stained with CD86 **(C)** or CD80 **(D)**, and analyzed by flow cytometry. Right: The relative mean fluorescence intensity (RMFI) of n = 7 patients are shown. Data were analyzed by two-way ANOVA **(D, E)**, or two-tailed paired Student’s t-test **(F, G)**. Data are presented as mean ± SD.

Defective monocyte-derived DC cells constitute a potential reason for the poor therapeutic efficacy of PEG-IFNα-2b (35). As CD64 has been identified to possess the capability to separate conventional DC cells from monocyte-derived DC cells (36), CD64^+^CD11c^+^ monocyte-derived DC cells on HBV patients were tested during PEG-IFNα-2b therapy. CD64^+^CD11c^+^DC cells were significantly decreased and remained at a very low level during PEG-IFNα-2b therapy, and increased after the PEG-IFNα-2b therapy stopped (**Figures 6A, B**). Combined with the dynamic changes of CD24^+^CD38^hi^ B cells, the changes of CD64^+^ Mo-derived DC cells were negatively related to the changes of CD24^+^ CD38^hi^B cells during PEG-IFNα-2b therapy (**Figure 6C**). To confirm whether Breg cells have suppression capability of mo-DCs cells, Mo-DC cells were sorted from HBV patients as CD19^-^CD3^-^CD11c^+^HLA-DR^+^cells and co-cultured with or without sorted Breg cells at 1:1 cell ratio. After cultured 24 h, the important markers CD86 and CD80 were tested. These data showed that Breg can decrease the expression of CD80 and CD86 expression on mo-DC cells and this effected can be blocked by anti-IL-10 antibody (**Figures 6D, E**). To further identify whether the regulatory function of Breg cells toward mo-DC is cell-contact dependent or not, we did the transwell experiment to physically block cell-cell contact between mo-DC and Breg cell. The results showed that breg cells still can re-modulate mo-DC phenotype showing that this regulation is not cell-contact dependent but triggered by the immunosuppressive cytokines (**Figures 6F, G**). Thus, CD24^+^CD38^hi^ B cells inhibit the functions of anti-virus immune system and result in immune suppression during Peg-IFNα therapy, which confirms that the immunomodulatory effects of PEG-IFNα-2b exist in human.

### CD24 Is a Suitable Marker to Target CD24^+^CD38^hi^ B Cells

**Figure 7:**
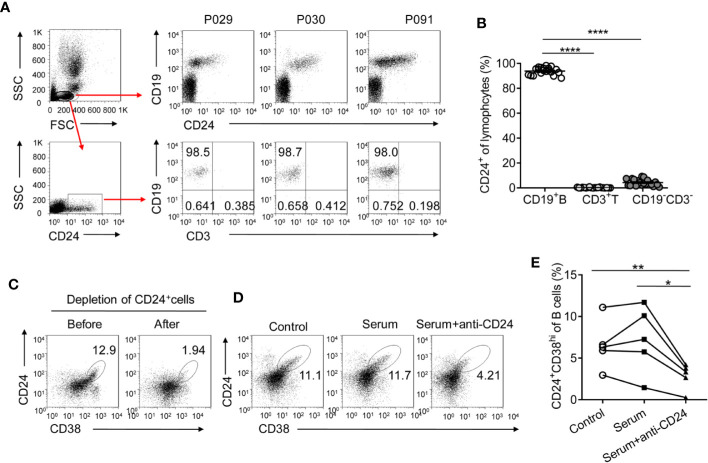
CD24 is a suitable target for CD24^+^CD38^hi^ B cell depletion. **(A)** Gating strategy and representative density plots of CD19 and CD24 expression on gated lymphocytes from HBV patients at 12 weeks (12w) after the initiation of PEG-IFNα-2b therapy. After gating on CD24^+^ lymphocytes, nearly 95% of cells were CD19^+^CD3^-^ B cells. **(B)** Percentage analysis of gated CD24^+^ cells belonged to CD19^+^ B, CD3^+^ T cell, and CD19^-^CD3^-^ other cell populations. n = 20. Paired two-tailed t-tests. **(C)** Representative density plots showing CD24 and CD38 expression on gated lymphocytes from HBV patients before and after CD24^+^ cells were sorted. CD24^+^ cells were sorted using an anti-CD24 antibody (BD Bioscience) and anti-FITC-MicroBeads (MiltenyiBiotec). **(D**, **E)** Representative density plots and percentage analysis of CD24 and CD38 expression in each group. PBMCs from HBV patients were cultured at a density of 1 × 10^6^ cells per 1 ml with 10 µg of anti-human CD24 antibody and 500 µl of guinea pig serum, with 500 µl of guinea pig serum only, or with 500 µl of complete RPMI medium 1640 (control) for 30min. n = 5. Paired two-tailed t-tests Mean ± SEM, *p < 0·05, **P < 0·01, ****P < 0·0001.

Given that CD24^+^CD38^hi^ B cells are significantly induced during PEG-IFNα-2b therapy, the strategies to selectively manipulate this opposing activity would be prospected to restore immune responses and improved persistent virus therapy. Anti-CD19 and anti-CD20 antibodies have previously been used to deplete B cells (37, 38). However, such treatments deplete all B cells, including CD27^+^CD38^hi^ precursors of plasma B cells and CD24^-^ mature B cells, which may be beneficial to the immune response. It is important to identify a target marker that is specifically expressed on CD24^+^CD38^hi^ B cells but not on other B cell subsets and effector cells. We analyzed blood from HBV patients at 12 weeks after the start of Peg-IFNα-2b therapy when CD24^+^CD38^hi^ B cells increased to very high levels. The CD24 surface marker was expressed primarily on CD19^+^ B cells but not on other effector immune cells, including T cells and NK cells, making CD24 a suitable target for CD24^+^CD38^hi^ B cell depletion (**Figures 7A, B**). As approximately 95% of CD24^+^ lymphocytes were B cells (**Figures 7B**), the first thing we do is to sorting CD24^+^ B cells from the lymphocytes of HBV patients using the anti-CD24 antibody to identify whether we could deplete CD24^+^CD38^hi^ B subsets using anti-CD24 antibody. After depleting CD24^+^CD38^hi^ B subsets using anti-CD24 antibody, the percentage of CD24^+^CD38^hi^ B cells decreased whereas other B cell subsets were maintained (**Figure 7C**). To determine whether the anti-CD24 antibody depleted CD24^+^ cells through complement dependent cytotoxicity and to simulate the situation *in vivo*, PBMCs from HBV patients were cultured with guinea pig serum with or without the anti-CD24 antibody. PBMCs co-cultured with the anti-CD24 antibody and the guinea pig serum showed significantly decreased percentages of CD24^+^CD38^hi^ B cells compared with the guinea pig serum control group and the medium-only control group (**Figures 7D, E**). These results indicated the feasibility of using the anti-CD24 antibody to deplete CD24^+^CD38^hi^ B cells without harming other B cell subsets, suggesting a promising strategy to improve the therapeutic effects of Peg-IFNα-2b during HBV persistence.

## Discussion

PEG-IFNα-2b has been widely applied in many diseases yet yields disappointing therapeutic effects in treating chronic HBV infection. In this report, we have identified that PEG-IFNα-2b therapy may also induce an immunomodulatory effect in chronic HBV patients through dramatically upregulating the CD24^+^CD38^hi^ B cells, which drive an immunosuppressive program and reduce anti-virus therapeutic effects. The extent to which CD24^+^CD38^hi^ B cells were retained in a given patient was negatively correlated with therapeutic effects. In this context, CD24 was found to be a suitable marker to target CD24^+^CD38^hi^ B cells. Given that Type I IFNs exert diverse effects on innate and adaptive immune cells and have been widely employed to treat infection with viruses, bacteria, fungi, parasites and tumors (2, 8), the findings presented here are relevant not only for understanding HBV but also in ascertaining the role of immunomodulatory effect induced by the widely used PEG-IFNα-2b therapy.

Although early antiviral effects of IFN-α are critical, the potential immunomodulatory roles of IFNs later in chronic infection could explain paradoxical clinical observations using IFN-α based treatments. For example, IFN-α warrants the survival of the host in the acute phase of infection (39), while persistent IFN-α-induced inflammation may also paradoxically promote microbial evasion during chronic infection (11, 12). IFNα has been reported to lead to the production of immunosuppressive molecules in models of Simian Immunodeficiency Virus (SIV) (39) and LCMV infection (11, 12). In addition, IFNα reduces the responsiveness of macrophages to activation by IFN*γ* during *Listeria monocytogenes* and *Mycobacterium tuberculosis* infections (40–42). Data presented here confirms the effect of Peg-IFNα therapy to induce immunosuppressive CD24^+^CD38^hi^ B cells in chronic infection. Interestingly, both the percentage and number of CD24^+^CD38^hi^ B cells were at high level at 12–24 weeks after the beginning of Peg-IFNα therapy and then decline. This decline of CD24^+^CD38^hi^ B cells may because long-term high concentrations of IFN-α overexposure. Type I IFN therapy in chronic HCV patients may result in lupus-like symptoms (43). In lupus patients, hyperactivated pDCs fail to induce Breg cells (30). It is possible that the chronic high concentrations of type I IFN is potential to induce autoimmune-like disease rather than generates CD24^+^CD38^hi^ Breg cell at the late stage of the therapy. The specific mechanism of the Breg decline after long-term stimulation of IFNα is still unknown. It is also important to identify the molecular basis for the antiviral versus immunomodulatory effects of IFN-α to selectively manipulate these opposing activities.

Previous reports have showed CD24 is a potential oncogene and overexpressed in a large variety of human malignancies (44). Many efforts have been done for early intervention about CD24 target in the prevention and treatment of cancer, such as colorectal cancer, pancreatic cancer (45), ovarian cancer (46) and bladder cancer (47). It seems promising that the therapeutic potential of CD24 blockade with monoclonal antibodies, which may promote the phagocytic clearance of CD24^+^ cancer cells both *in vitro* and *in vivo* (20). In this study, CD24^+^CD38^hi^ B cells was observed along with subsequent negative correlation with monocyte-derived DC cells, NK cells, and T cells functions in HBV patients (**Figures 5** and **6**). It was potential that depleting CD24^+^B cells using an anti-CD24 antibody would improve Peg-IFNα therapy against the virus *in vivo*. Nevertheless, more evidences about anti-CD24 antibody based on clinical studies are urgently needed.

In conclusion, Peg-IFNα-2b therapy is the most effective HBV therapy and yet is only capable of inducing an HBeAg seroconversion rate of approximately 30%. The study presented here uncovers the mechanism by which a CD24^+^CD38^hi^ B-centered immunomodulatory response is induced by persistent Peg-IFNα-2b therapy. In addition, we demonstrate possible effective strategies to interrupt the immunosuppressive state using an anti-CD24 antibody. Other approaches which selectively manipulate these opposing activities between immune-activation and immunomodulation may also be possible to increase the anti-viruses immune response.

## Data Availability Statement

The original contributions presented in the study are included in the article/**Supplementary Material**. Further inquiries can be directed to the corresponding authors.

## Ethics Statement

The studies involving human participants were reviewed and approved by the local Ethics Board of the First Affiliated Hospital of Anhui Medical University. The patients/participants provided their written informed consent to participate in this study.

## Author Contributions

BF designed and performed the experiments, analyzed and interpreted the data. DW performed the experiments and assessing outcomes. XS and CG helped to perform the experiments. RS established techniques of FACS and Immunohistochemistry, and interpreted the data. YL and YY generated the random allocation sequence, enrolled participants, collected tissue samples and information from patients. JL supervised the clinical trial and assigned participants to interventions. ZT provided strategic planning, conceived the project, and interpreted some data. HW supervised the project, provided crucial ideas, and assisted with data interpretation. BF wrote the manuscript with HW. All authors contributed to the article and approved the submitted version.

## Funding

This work was supported by the Ministry of Science and Technology of China (973 Basic Science Project 2012CB519004), the Natural Science Foundation of China (81330071, 81922028), Anhui Key Program of Medical Scientific Research of China (no.2010A010), and Youth Innovation Promotion Association of Chinese Academy of Sciences (grant 2019442).

## Conflict of Interest

The authors declare that the research was conducted in the absence of any commercial or financial relationships that could be construed as a potential conflict of interest.

## Abbreviations

HBV, hepatitis B virus; IFNα, Interferon-α TH1, T helper 1 cells; TH17, T helper 17 cells; CHB, Chronic Hepatitis B; HBeAg, Hepatitis B e antigen; LCMV, lymphocytic choriomeningitis virus; IFN-I, Type I interferon; Breg, regulatory B.
