# Supplementary material for: Immunomodulation Induced During Interferon-α Therapy Impairs the Anti-HBV Immune Response Through CD24+CD38hi B Cells
Source: Front Immunol. 2020 Dec 23;11:591269. doi: 10.3389/fimmu.2020.591269 (PMC7786281; doi:10.3389/fimmu.2020.591269)
Supplement: Supplementary file 1 [file DataSheet_1.docx]

**Supplementary Materials and Methods**

**Extracellular and intracellular** **flow cytometry staining**

Lymphocyte suspensions were stained with the human/mouse monoclonal antibodies described in STable 3. Homologous IgGs were used as negative control Abs. Fluorescence-activate cell sorting (FACS) staining was performed according to the manufacturer’s instructions (BD Biosciences). Data from 20,000–50,000 single-cell events were collected using a standard FACS Calibur flow cytometer (BD Biosciences). Intracellular staining was performed on human or mouse mononuclear cells after 4 h of stimulation with PMA (50 ng/mL; Sigma) and ionomycin (1 μg/mL; Calbiochem) in the presence of monensin (10 μg/mL; Sigma). The cells were then collected, washed, and blocked (Foxp3/Transcription Factor Staining Buffer Set, eBioscience).

**Co-culture system with or without CD24^+^CD38^hi^ B cells**

The PBMCs of CHB patients were cultured in 24-well ﬂat-bottom plates at a density of 1-2 × 10^6^ cells per well in a complete RPMI medium 1640 (Gibco, Grand Island, NY, U.S.A.) with 10% fetal bovine serum (HyClone, Logan, UT, U.S.A.) plus streptomycin and penicillin as well as IL-2 (100 U/ml; Jiangsu Kingsley Pharmaceutical Co., Ltd., China). PBMC samples have been mock sorted by FACS sorting method and each lymphocyte subset including T, NK and B cells have first been analysed to show that sorting method itself would not effect on the percentage of lymphocyte subsets (Data not shown). PBMC from peripheral blood mononuclear cells of CHB patients with or without CD19^+^CD24^+^CD38^hi^ B cells were sorted (BD Bioscience) and the purity of depleting CD19^+^CD24^+^CD38^hi^ B cells was determined to be >95% by post-purification FACS analysis. Cell purity were stimulated with 50μg/ml HBsAg (HyTest, 8HS7ay) for 72 hours and cytokines were measured by intracellular staining.

**In vitro IFNa stimulations**

PBMCs from HBV patients and healthy controls were cultured in complete RPMI medium 1640 with or without human Peg-IFNα-2b (100ng/mL) for 36h. PBMCs from CHB patients were collected from patients with Peg-IFNα-2b treatment for 12-36 weeks. The percentage of CD24^+^CD38^hi^ B cells were evaluated by extracellular flow cytometry staining. Lymphocyte suspensions were stained with the human/mouse monoclonal antibodies described in STable 3. Homologous IgGs were used as negative control Abs.

**The suppression of mo-DCs**

To analyze the suppression of mo-DCs by Bregs, we first sorted mo-DCs (gated by CD3^-^CD19^-^ HLADR^+^CD11c^+^CD64^+^) and Breg cells (gated by CD3^-^CD19^+^ CD24^+^CD38^high^), performed using a FACS Aria (BD Bioscience) from the the peripheral blood of CHB patients. The purity of these sorted populations was greater than 92%. To analyze the suppression of mo-DCs by Bregs, and whether it is triggered by the release of IL-10, mo-DCs were cultured in 48-well ﬂat-bottom plates at a density of 0.5-1.0 × 10^5^ cells per well in a complete RPMI medium 1640 (Gibco, Grand Island, NY, U.S.A.) with 10% fetal bovine serum (HyClone, Logan, UT, U.S.A.) plus streptomycin and penicillin. Equal number of Breg cells were then added to the culture wells, with or without 3 μg/ml anti-IL-10 (biolegend; #501427) for 24 hours.

Besides, to analyze the suppression of mo-DCs by Bregs, and whether it is cell-contact dependent, mo-DCs were cultured on the top chamber of the transwell system, and Breg cells were cultured on bottom chamber of transwell plates. After 24 hours, cells were then collected and stained in accordance with the manufacturer's instructions, using antibodies purchased from BD Biosciences. Data were collected using the FCM LSR II flow cytometer (BD Biosciences, USA) and analyzed with FlowJo software (Tree Star, USA).

**Enzyme-Linked Immunosorbent Assay**

ELISA analysis of IL-10 secretion in cell-free supernatants of cultured PBMCs from HBV patients and healthy controls with or without re-stimulation by Peg-IFNα (25ng/mL) during a 32-h culture. PBMCs from HBV patients at 0 week or 12-36 weeks after the start of IFNα therapy were cultured in 24-well flat-bottom plates at a density of 1-2×10^6^ cells per well in a complete RPMI medium 1640 with 10% fetal bovine serum plus streptomycin and penicillin as well as IL-2 (100 U/ml). Cell culture supernatant was firstly collected and centrifuged at 4°C, 16,000 g for 10 min to remove cell fragments.IL-10 enzyme-linked immunosorbent assay (ELISA) kit was purchased from Dakewe (Dakewe Biotech Co., Ltd. China). For IgG ELISA analysis, PBMC with or without CD19^+^CD24^+^CD38^hi^ B cells were re-stimulated with 50μg/ml HBsAg (HyTest, 8HS7ay) for 72 hours and cell-free supernatants of cultured PBMCs in each group were measured by ELISA. IgG ELISA kits were purchased from [MultiSciences](http://www.liankebio.com/brand-MultiSciences.html). Experiments were carried out according to the manufacturer’s instructions. To demonstrate wheher increase numbers of immature B-cells is a compensatory mechanism with accelerated emigration from BM or spleen, we tested the circulating levels of BAFF (Multisciences; 70-EK1102-96), CXCL12 (Multisciences; 70-EK1119-96), and CXCL13 (Multisciences; 70-EK1105-96) of HBV patients at 12-36 weeks after the start of IFN- α therapy by ELISA.

**Statistical analyses**

We performed unpaired two-tailed t-tests or paired two-tailed t-tests (difference between two groups) to determine statistical significance. Differences between rates were tested with the χ^2^ or Fisher’s exact test, if appropriate. All p-values were two-tailed and were considered significant when less than 0·05. The Statistical analyse used in each Figure has been specifically described in each Figure legend.

**Supplementary Figures and Figure Legends**


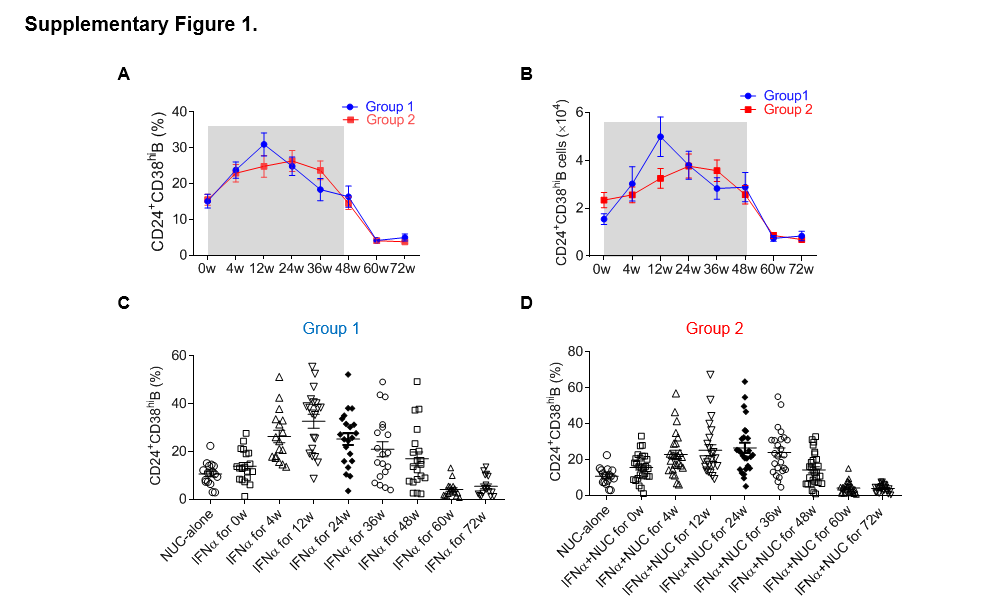


Supplementary Figure 1. CD24^+^CD38^hi^ B cells increase significantly during Peg-IFNα-2b therapy.

(A) Percentage analysis of CD24^+^CD38^hi^ B cells from HBV patients during Peg-IFNα therapy in Group 1 (Blue, n=20) and Group 2 (Red, n=27).

(B) Cell number of CD24^+^CD38^hi^ B cells from HBV patients during Peg-IFNα therapy in Group 1(Blue, n=20) and Group 2 (Red, n=27).

(C) Percentage analysis of CD24^+^CD38^hi^ B cells from HBV patients using NUC-alone therapy (n=17), before IFNα treatment (0w) and at each time points during Peg-IFNα therapy (n=18~20). Unpaired t test.

(D) Percentage analysis of CD24^+^CD38^hi^ B cells from HBV patients using NUC-alone therapy (n=17), before IFNα+NUC treatment (0w), and at each time points during Peg-IFNα+NUC therapy (n=18~20). Unpaired t test.


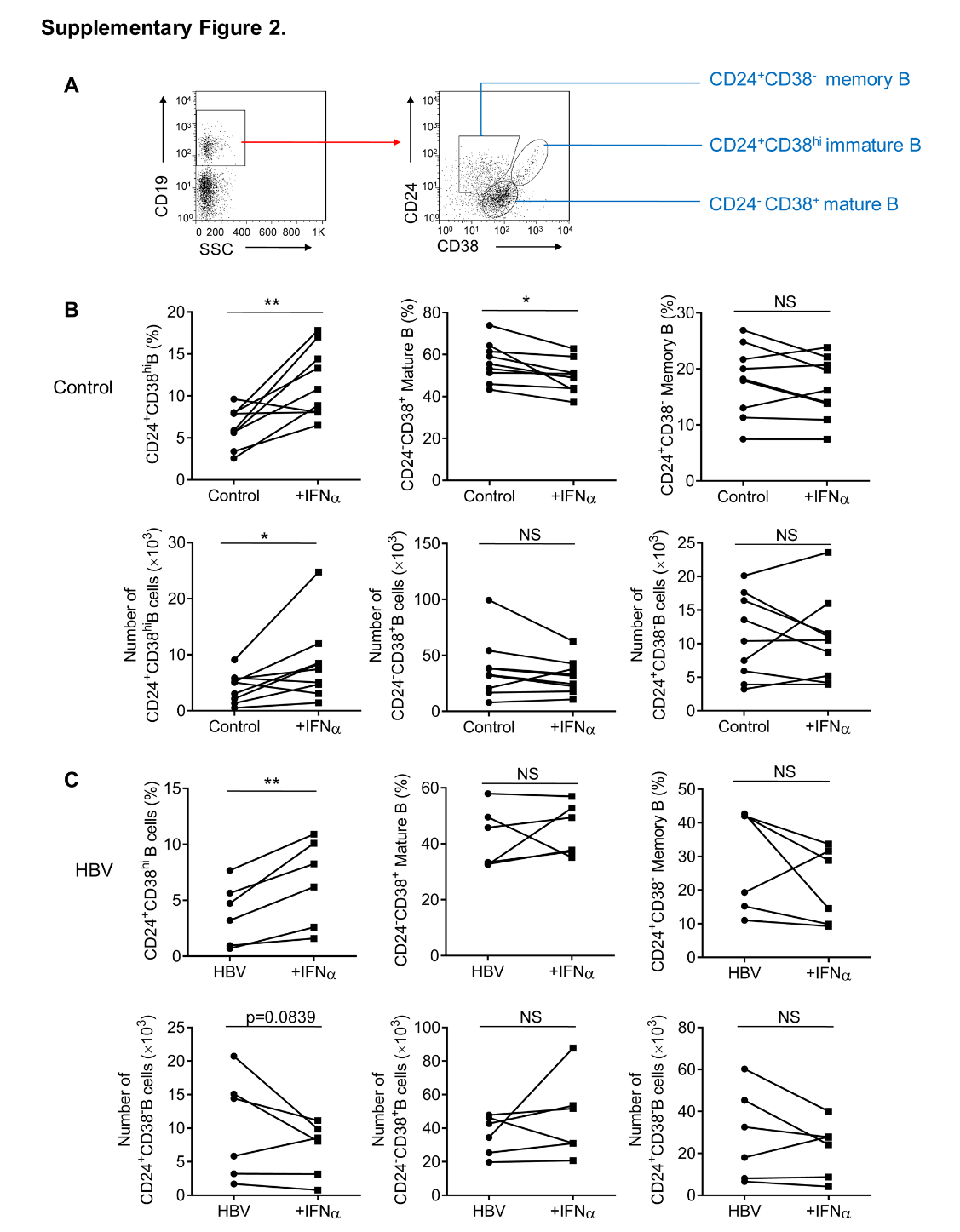


Supplementary Figure 2. Dynamic change of B cell subsets induced by Peg-IFNa-2b.

PBMCs from HBV patients and healthy controls were cultured in complete RPMI medium 1640 with or without human Peg-IFNα (100ng/mL) for 36h.

1. Gating strategy of B cell subsets.
2. Percentage analysis showing the change of percentage and number of each B cell subset in healthy controls. n=9. Paired t test.
3. Percentage analysis showing the change of percentage and number of each B cell subset in HBV patients. n=6. Paired t test.

Mean ± SEM, *p<0·05, **P<0·01, ***P<0·005, ****P<0·0001

Supplementary Figure 3.


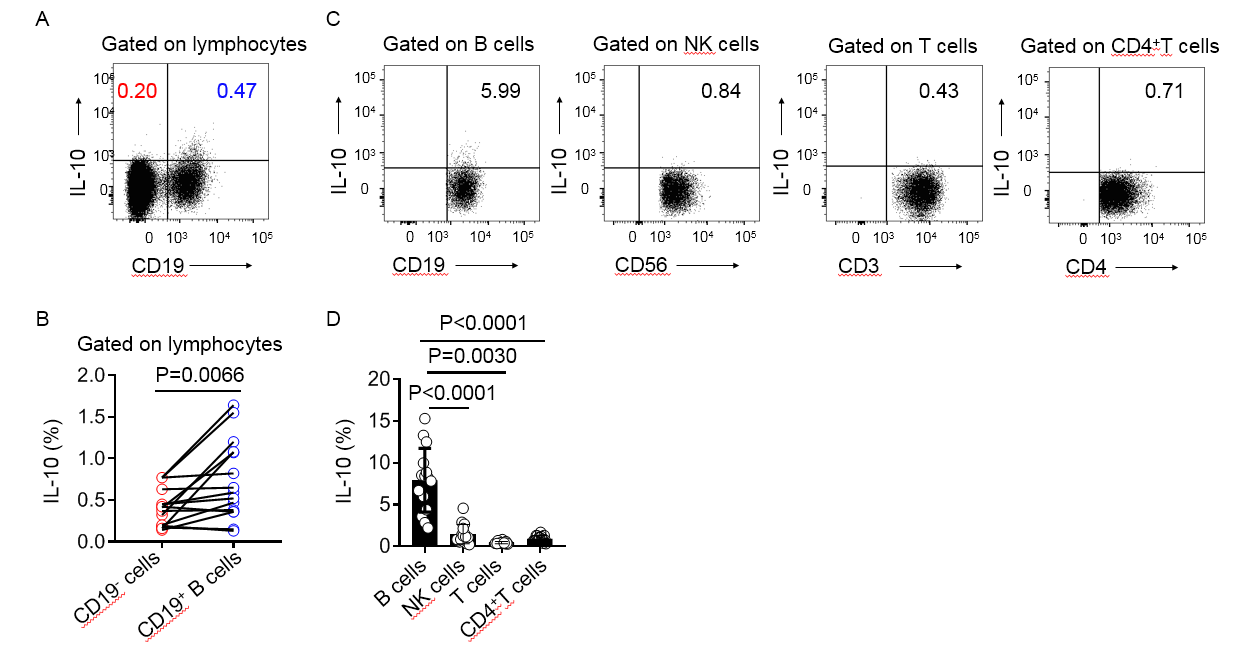


Supplementary Figure 3. IL-10 expression mainly in B cells.

(A) Flow cytometry analysis of IL-10 expression in CD19^+^ B cells and CD19^-^ cells gated from lymphocytes of PBMC from CHB patients with IFN-α therapy for 12-36 weeks.

(B) Statistics calculated by the percentage of IL-10^+^ cells in CD19^+^ B cells (blue) and CD19^-^ cells (red) gated from lymphocytes of PBMC from CHB patients with IFN-α therapy for 12-36 weeks. n = 15. Paired t test. *P < 0.05; **P < 0.01; ***P < 0.001; ****P < 0.0001. Data are presented as mean ± SD.

(C) Flow cytometry analysis of IL-10 expression on B cells, NK cells, T cells and CD4^+^ T cells from PBMC of CHB patients with IFN-α therapy for 12-36 weeks.

(D) Statistics calculated by the percentage of IL-10^+^ cells in B cells, NK cells, T cells and CD4^+^ T cells from PBMC of CHB patients with IFN-α therapy for 12-36 weeks. n = 15. Data were analyzed by two-way ANOVA. *P < 0.05; **P < 0.01; ***P < 0.001; ****P < 0.0001, and presented as mean ± SD.


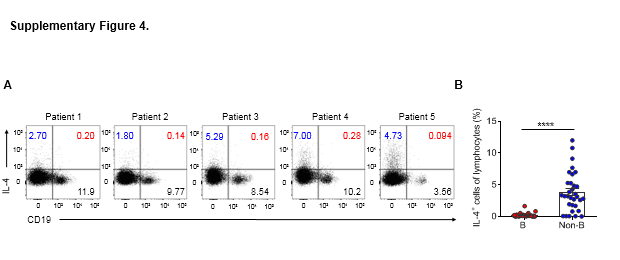


Supplementary Figure 4. No significant IL-4 secreted by B cells from HBV patients during Peg-IFNα therapy.

PBMC from HBV patients were collected after 3-9 month of the beginning of Peg-IFNα therapy. Intracellular flow cytometry staining was done according to the experimental procedures.

(A) Representative density plots showing IL-4 and CD19 expression in gated CD45^+^ cells from HBV patients during Peg-IFNα therapy.

(B) Percentage analysis of IL4^+^ cells in lymphocytes from HBV patients during Peg-IFNα therapy. n = 32. Mean ± SEM, *p<0·05, **P<0·01, ***P<0·005, ****P<0·0001


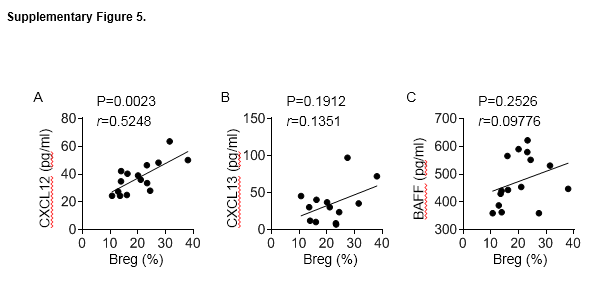


Supplementary Figure 5. The correlation of the percentage of Breg cells and BAFF, CXCL12, and CXCL13 levels in serum of CHB patients.

(A-C) PBMC and serum were collected from HBV patients at 12-36 weeks after the start of IFN-α therapy. Spearman’s rank correlation coefficient comparing the percentage of Breg cells and the levels of the indicated molecules. n = 15. The Spearman correlation coefficient (r) and P value are shown.


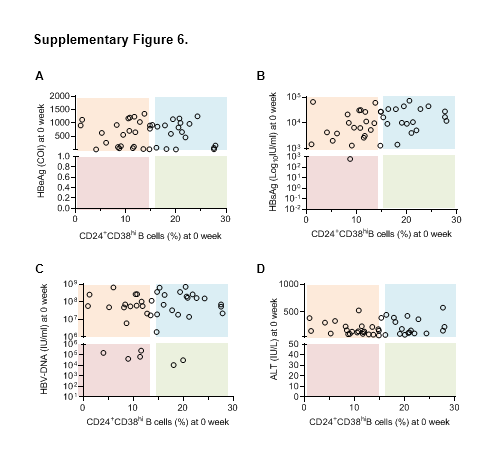


Supplementary Figure 6. No significant differences in patients with fewer or more CD24^+^CD38^hi^ B cells before Peg-IFNα therapy.

(A) Analysis between the percentage of CD24^+^CD38^hi^ B cells in patients at 0 week and HBeAg expression at 0 weeks after the start of Peg-IFNα therapy. n=42

(B) Analysis between the percentage of CD24^+^CD38^hi^ B cells in patients at 0 week and HBsAg expression at 0 weeks after the start of Peg-IFNα therapy. n=42

(C) Analysis between the percentage of CD24^+^CD38^hi^ B cells in patients at 0 week and HBV-DNA level at 0 weeks after the start of Peg-IFNα therapy. n=42

(D) Analysis between the percentage of CD24^+^CD38^hi^ B cells in patients at 0 week and ALT level at 0 weeks after the start of Peg-IFNα therapy. n=42. Each circle in the Figure represents the data from a patient.

**Supplementary Table 1. Clinical characteristics of the study patients.**

| **Characteristics** | **Group 1 (n=45)** | **Group 2 (n=47)** |
| --- | --- | --- |
| Demography |  |  |
| Median age, years (Range) | 29 (19-45) | 27 (18-41) |
| Male | 37 (82.2%) | 29 (61.7%) |
| HBV genotype |  |  |
| B | 20 (44.4%) | 24 (51.1%) |
| C | 25 (55.5%) | 22 (46.8%) |
| Others | 0 | 1 (2.13%) |
| Mother-to-fetus transmission | 15 (33.3%) | 18 (38.3%) |
| Laboratory results |  |  |
| Median HBeAg, log COI (Range) | 2.60 (0.9-3.1) | 2.31 (0.5-3.1) |
| Median HBV DNA, log IU/mL (Range) | 7.57 (4.0-10.2) | 7.30 (4.5-8.9) |
| Median HBsAg, log IU/mL (Range) | 4.00 (2.9-4.7) | 3.97 (1.6-4.9) |
| Median ALT, ULN (Range) | 204 (84-480) | 231 (80-580) |

*All subjects were HBsAg positive, HBeAg positive, and Chinese patients.

HBsAg, hepatitis B surface antigen; HBeAg, hepatitis B e antigen; ALT, alanine aminotransferase; ULN, upper limit of the normal range. Laboratory results referred to results tested before treatment (at week 0).

**Supplementary Table 2. Therapeutic outcome at the end of treatment (week 72).**

*HBeAg loss and seroconversion to anti-Hbe. P=0.5011 compared with Group 1 and Group 2 (Fisher’s exact test, Two tailed)

| Treatment Outcome | Group of Drugs | No. of Patients  at week 72 | % of Patients  at week 72 |
| --- | --- | --- | --- |
| Anti-HBeAg seroconversion* | Group1: PEG-IFNα | 12/45 | 26.7% |
|  | Group 2: PEG-IFNα+AVD | 16/47 | 34.0% |
|  | Group1+Group2 | 28/92 | 30.4% |

**Supplementary Table 3. Antibodies used in this study.**

| Antibody | Clone | Brand | Cat.No. |
| --- | --- | --- | --- |
| PerCP-CY5.5 Mouse Anti-Human CD3 | SK7 | BD Bioscience | 340949 |
| APC-CY7 Mouse Anti-Human CD3 | SK7 | BD Bioscience | 557832 |
| APC-Cy7 Mouse Anti-Human CD14 | MφP9 | BD Bioscience | 557831 |
| FITC Mouse Anti-Human CD16 | 3G8 | BD Bioscience | 555406 |
| FITC Mouse Anti-Human CD19 | HIB19 | BD Bioscience | 555412 |
| PE-CY7 Mouse Anti-Human CD19 | SJ25C1 | BD Bioscience | 557835 |
| FITC Mouse Anti-Human CD24 | ML5 | BD Bioscience | 555427 |
| FITC Mouse Anti-Human CD27 | M-T271 | BD Bioscience | 555440 |
| PerCP-CY5.5 Mouse Anti-Human CD38 | HIT2 | BD Bioscience | 551400 |
| FITC Mouse Anti-Human CD8 | RPA-T8 | BD Pharmingen | 555366 |
| FITC Mouse anti-human Lineage Cocktail | UCHT1 | Biolegend | 348801 |
| PE Mouse Anti-Human CD123 | 6H6 | Biolegend | 306006 |
| APC-CY7 Mouse Anti-Human CD11c | Bu15 | Biolegend | 337218 |
| APC Mouse Anti-Human HLA-DR | G46-6 | BD Bioscience | 559866 |
| 7-AAD |  | BD Bioscience | 559925 |
| PE-Cy7 Mouse Anti-Human CD64 | 10.1 | Biolegend | 305022 |
| APC Mouse Anti-Human CD107a | H4A3 | BD Bioscience | 560664 |
| Purified Mouse Anti-Human CD24 | ML5 | BD Pharmingen | 555426 |
| FITC Mouse Anti-Human IFN-γ | B27 | BD Bioscience | 554700 |
| PE Mouse Anti-Human IL-10 | JES3-9D7 | BD Bioscience | 559337 |
| PE Mouse Anti-Human TNF-α | MAb11 | BD Bioscience | 559321 |
| APC Mouse Anti-Human IL-4 | 8D4-8 | Biolegend | 500714 |
| Brilliant Violet 421 Mouse Anti-Human CD56 | HCD56 | Biolegend | 318328 |
| APC Annexin V |  | BD Bioscience | 550474 |
| Alexa Fluor® 647 Mouse Anti-Human Ki-67 | B56 | BD Bioscience | 561126 |
| PE FOXP3 Monoclonal Antibody | PCH101 | eBioscience | 12-4776-42 |
| FITC Mouse Anti-Human CD25 | M-A251 | BD Bioscience | :555431 |
| Brilliant Violet 421 Mouse Anti-Human CD86 | BU63 | Biolegend | 374212 |
| Brilliant Violet 510 Mouse Anti-Human CD80 | 2D10 | Biolegend | 305234 |
